# Refractory Celiac Disease: Nutritional Failure, Immune Dysregulation, and Lymphomagenesis

**DOI:** 10.3390/nu18152479

**Published:** 2026-07-31

**Authors:** Ioanna Aggeletopoulou, Ploutarchos Pastras, Maria Kalafateli, Christos Triantos

**Affiliations:** 1 Division of Gastroenterology, Department of Internal Medicine, University of Patras, 26504 Patras, Greece; ploutarchosp96@gmail.com (P.P.); chtriantos@upatras.gr (C.T.); 2 4th Department of Internal Medicine, Aristotle University of Thessaloniki, Hippokratio Hospital, 54642 Thessaloniki, Greece; mkalafaa@auth.gr

**Keywords:** refractory celiac disease, gluten-free diet, nutritional status, intraepithelial lymphocytes, interleukin-15, JAK/STAT signaling, clonal evolution, enteropathy-associated T-cell lymphoma

## Abstract

Refractory celiac disease (RCeD) is a rare but severe complication of celiac disease characterized by persistent or recurrent malabsorptive symptoms and villous atrophy despite a strict gluten-free diet, after exclusion of ongoing gluten exposure, alternative enteropathies, and overt lymphoma. RCeD comprises two biologically distinct entities. RCeD-I is associated with phenotypically normal, polyclonal intraepithelial lymphocytes and generally reflects persistent gluten-independent mucosal inflammation with a relatively favorable prognosis. RCeD-II is defined by expansion of aberrant clonal intraepithelial lymphocytes lacking normal surface T-cell markers and is increasingly regarded as a low-grade intraepithelial lymphoma or in situ lymphomatous disorder, with substantial risk of progression to enteropathy-associated T-cell lymphoma (EATL). Mechanistic studies identify epithelial stress, IL-15-driven IEL survival, stromal and innate immune amplification, and cytotoxic epithelial injury as central drivers of refractory mucosal damage. In RCeD-II, aberrant IELs acquire a hybrid T/NK-like phenotype, persist through anti-apoptotic IL-15/JAK–STAT signaling, and induce enterocyte killing, while molecular alterations involving JAK1, STAT3, JAK/STAT regulators, NF-κB signaling, epigenetic regulators, and chromosomal abnormalities support stepwise lymphomagenesis. Recent single-cell multiomic studies further reveal genetically altered intestinal lymphocyte clones and intratumoral heterogeneity across the RCeD-II–EATL continuum. From a nutritional immunology perspective, RCeD illustrates a setting in which removal of the initiating dietary antigen is insufficient to restore mucosal immune homeostasis. This review summarizes the pathogenic processes that distinguish RCeD-I from RCeD-II and link failed mucosal recovery after gluten withdrawal to persistent immune-mediated epithelial injury, aberrant IEL expansion, clonal evolution, and lymphoma progression.

## 1. Introduction

Celiac disease (CeD) is a chronic, immune-mediated enteropathy triggered by the ingestion of gluten-containing proteins in genetically susceptible individuals [1,2,3]. It affects approximately 1% of the general population, particularly in Western countries, and represents one of the most common systemic autoimmune conditions of the gastrointestinal tract [4]. CeD can develop and be diagnosed at any age, although its incidence is higher in children than in adults. In young children, the classical clinical presentation typically emerges after the introduction of gluten-containing foods [5,6,7]. Genetic predisposition plays a central role in disease susceptibility, as almost all patients bear the human leukocyte antigen (HLA) molecules HLA-DQ2 and/or HLA-DQ8, while rare HLA-DQ2/DQ8-negative patients may be associated with HLA-DQ7.5, which represents less than 1% of patients with CeD [8].

The clinical spectrum of CeD is significantly heterogeneous. Classical manifestations include diarrhea, weight loss, anemia, micronutrient deficiencies, abdominal pain, and impaired growth in children, primarily reflecting impaired nutrient absorption due to villous atrophy [9]. However, many patients present with non-classical or extraintestinal features, including neurological symptoms, endocrine disorders, osteopenia, cutaneous manifestations, infertility, and other reproductive disturbances [10]. In selected cases, CeD may remain clinically silent and be detected only through serological screening. Long-standing or untreated disease may lead to severe complications, including small bowel adenocarcinoma and enteropathy-associated T-cell lymphoma (EATL), an aggressive peripheral T-cell lymphoma arising in the setting of chronic intestinal immune activation [11].

The diagnosis of CeD is based on an integrated assessment of serological and histological findings. HLA-DQ2/DQ8 typing has a selective role in diagnostically uncertain cases because of its high negative but low positive predictive value [2]. Serologic testing is central to the diagnostic workup of CeD and should be executed while the patient is consuming a gluten-containing diet [2]. In adults, IgA anti-tissue transglutaminase 2 antibodies (anti-TG2/anti-tTG), together with total serum IgA measurement, are recommended as the first-line serologic approach [2]. IgA anti-endomysial antibodies (EMA), although highly specific, are not routinely required for confirmation but may be reserved for diagnostically unclear cases. In patients with selective IgA deficiency, IgG-based assays, such as IgG anti-TG2 or IgG anti-deamidated gliadin peptide antibodies (IgG anti-DGP), should be used [2].

When histological confirmation is required, current adult guidelines recommend obtaining at least four biopsy specimens from the distal duodenum and two from the duodenal bulb, even in the presence of normal endoscopic appearance, because celiac lesions may be patchy [2]. Biopsies should be adequate and well oriented, allowing assessment of intraepithelial lymphocytosis, villous architecture, villous atrophy, and crypt hyperplasia, and interpreted using the modified Marsh classification [2,12]. In selected adults under 45 years with IgA anti-TG2 titers ≥ 10 times the upper limit of normal, confirmed in a second blood sample, a no-biopsy diagnostic approach may be considered [2].

In children, ESPGHAN 2020 guidelines allow a no-biopsy diagnosis of CeD when IgA anti-TG2 titers are ≥10 times the upper limit of normal and EMA-IgA positivity is confirmed in a second blood sample [7]. If histological confirmation is needed, at least four biopsy specimens from the distal duodenum and at least one from the duodenal bulb should be collected while the child remains on a gluten-containing diet [7].

At present, the only established treatment for CeD is lifelong strict adherence to a gluten-free diet (GFD) [3,13]. Although most patients improve clinically and serologically, mucosal recovery may be delayed, particularly in adults [3,13]. Persistent or recurrent symptoms and/or villous atrophy despite a presumed strict GFD define the clinical scenario of non-responsive CeD, which requires systematic evaluation before refractory CeD (RCeD) is assumed [14]. RCeD represents a rare but severe subgroup within this spectrum, characterized by persistent malabsorptive symptoms and villous atrophy despite strict gluten avoidance after exclusion of ongoing gluten exposure and alternative causes of enteropathy [3,13].

The pathogenesis of CeD is driven by an abnormal immune response to gluten peptides in genetically susceptible individuals. Following gluten ingestion, tissue TG2 deamidates gluten-derived peptides, increasing their affinity for HLA-DQ2 or HLA-DQ8 molecules on antigen-presenting cells [1,15]. This promotes activation of gluten-specific CD4^+^ T cells in the intestinal lamina propria, leading to cytokine-mediated inflammation and epithelial damage [1,15,16]. The hallmark of active CeD is small intestinal mucosal injury, characterized by increased intraepithelial lymphocytes (IELs), crypt hyperplasia, and variable degrees of villous atrophy [1,15]. These immune-mediated alterations underlie malabsorption, nutritional deficiencies, and systemic complications.

RCeD is defined by persistent or recurrent symptoms of malabsorption and villous atrophy despite at least 12 months of a strict GFD, after exclusion of gluten contamination and alternative causes of enteropathy, including overt lymphoma [1,17,18]. RCeD occurs almost exclusively in adults and accounts for only a small proportion of patients with non-responsive CeD. Its diagnosis is challenging and requires confirmation of the original diagnosis of CeD, assessment of dietary adherence, histological re-evaluation, HLA typing, and detailed characterization of IELs [17].

This review aims to provide an updated overview of the immune mechanisms underlying RCeD, focusing on the pathways that maintain intestinal inflammation and drive aberrant IEL expansion despite strict GFD. By integrating recent advances in mucosal immunology, lymphocyte biology, nutritional immunology, and celiac-associated lymphomagenesis, we discuss the pathogenic basis of RCeD type I and type II and the biological continuum linking RCeD type II to EATL. Particular emphasis is placed on how failure of dietary antigen exclusion reveals persistent gluten-independent immune activation, epithelial stress, and clonal lymphocyte evolution.

## 2. Nutritional and Dietary Dimensions of Refractory Celiac Disease

RCeD is highly relevant to nutritional immunology because it arises in an autoimmune enteropathy whose primary treatment is dietary antigen exclusion [14]. In CeD, the GFD functions not only as nutritional treatment but also as the principal immune-modulating intervention, removing the dietary antigen that initiates adaptive and innate immune activation [1,15,19]. In uncomplicated CeD, a strict GFD removes the initiating dietary trigger and usually promotes clinical, serological, and eventually histological improvement [20]. Therefore, in patients with persistent symptoms or villous atrophy despite a presumed strict GFD, careful reassessment of dietary adherence, hidden gluten exposure, and cross-contamination is essential before true refractoriness is diagnosed [21]. When available, gluten immunogenic peptides in stool or urine may provide objective evidence of recent gluten exposure and help distinguish ongoing dietary antigen stimulation from true refractory disease [22,23].

Beyond gluten exposure, persistent enteropathy has major nutritional implications, particularly in RCeD-II and EATL, where severe malnutrition and malabsorption may already be present at diagnosis [24]. Nutritional deterioration, reflected by hypoalbuminemia, micronutrient deficiencies, weight loss, anemia, and severe malabsorption and malnutrition, are not merely clinical outcomes but also important indicators of disease activity and severity [20,24]. Nutritional status should therefore be interpreted together with histological severity, biochemical abnormalities, IEL phenotype, and TCR clonality during risk stratification [24,25].

From this perspective, RCeD illustrates the limits of dietary immune modulation; once epithelial stress, innate immune activation, interleukin 15 (IL-15)–driven IEL survival, and clonal IEL expansion become established, removal of the initiating dietary antigen may no longer be sufficient to restore mucosal immune homeostasis [14,26,27,28,29,30]. Persistence of villous atrophy and malabsorptive symptoms despite strict dietary gluten exclusion suggests that, in RCeD, mucosal immune injury may become partially autonomous from the original dietary trigger [14,17,30].

Accordingly, nutritional management should accompany immunological assessment throughout the course of RCeD. A detailed evaluation should include recent weight loss, anthropometric measurements, dietary intake, serum albumin, and targeted investigation of macro- and micronutrient deficiencies [3]. Identified deficiencies should be corrected individually through oral supplementation and, when clinically indicated, enteral nutritional support [3]. Common nutritional deficiencies, particularly those involving iron, folate, vitamin B12, and vitamin D, should be assessed and corrected according to laboratory findings and individualized dietary evaluation. Calcium and phosphate status should also be monitored, and calcium intake optimized when inadequate [3]. Elemental diets have shown potential clinical and histological benefit in small studies of RCeD-I but are not established as routine treatment [31]. By contrast, probiotic, prebiotic, and short-chain fatty acid-directed interventions remain investigational, with insufficient evidence to support their routine use in RCeD [32]. Parenteral nutrition may be considered in patients with severe malnutrition due to profound malabsorption [3]. Nutritional status should be reassessed during follow-up together with the clinical, biochemical, and histological response [3].

## 3. From Non-Responsive CeD to Refractory Disease: Diagnostic Prerequisites and Biological Classification

In most patients, GFD use induces clinical improvement, serological normalization, and gradual mucosal healing. However, the pace of recovery is variable [13]. Symptoms usually improve within weeks to months, whereas complete histological recovery, particularly in adults, may require several years and is not achieved in all patients [3,19]. Persistent or recurrent symptoms after 6 to 12 months on a presumed strict GFD are commonly referred to as non-responsive CeD (NRCeD), a broad clinical scenario that requires systematic evaluation rather than immediate classification as well-defined refractory disease [14]. Available evidence suggests that a substantial proportion of adults with CeD may fall within the NRCeD spectrum, although prevalence estimates vary considerably according to the follow-up duration, the exact definition used, and the clinical setting [33]. Importantly, NRCeD should be viewed less as a distinct disease entity and more as a diagnostic prompt to identify the specific cause of persistent symptoms or mucosal damage [17]. Only a small proportion of patients initially categorized as NRCeD ultimately fulfill criteria for true RCeD, highlighting the importance of this distinction to avoid premature labeling and unnecessary escalation of therapy.

The most common cause of NRCeD is ongoing gluten exposure, either intentional or inadvertent. Intentional non-adherence may reflect complex behavioral, educational, socioeconomic, or symptom-related factors, whereas inadvertent exposure is frequently related to hidden gluten, cross-contamination, or misconceptions about the GFD [33,34]. Other potential causes should also be excluded, including slow-responsive CeD, RCeD, initial misdiagnosis, functional gastrointestinal disorders, microscopic colitis, inflammatory bowel disease, bile acid diarrhea, exocrine pancreatic insufficiency, irritable bowel syndrome, medication effects, other autoimmune diseases, and malignant complications such as EATL or small-bowel adenocarcinoma [3,13]. Slow-responsive CeD represents a particularly important diagnostic consideration, as these patients ultimately improve but may require a prolonged period for clinical and histological recovery before more aggressive approaches are pursued [17]. Therefore, true refractoriness should be considered only after dietary causes, diagnostic uncertainty, and alternative or concomitant disorders have been carefully excluded.

RCeD is diagnosed only after confirmation of the original diagnosis of CeD, careful assessment of dietary adherence, exclusion of ongoing gluten exposure and alternative causes of enteropathy, and histological confirmation of persistent villous atrophy despite a strict GFD [17]. Clinically, RCeD may also be described according to its temporal pattern as primary, when no initial clinical or histological response to a GFD is achieved, or secondary, when malabsorptive symptoms and villous atrophy recur following an initial clinical and histological response [17]. Detailed characterization of IELs is then essential, as it forms the basis for the classification of RCeD into two biologically and clinically distinct entities, the type I refractory celiac disease (RCeD-I) and type II refractory celiac disease (RCeD-II) [35]. Importantly, T-cell receptor (TCR) clonality should not be interpreted per se, but in combination with IEL immunophenotype, histological damage, clinical severity, biochemical abnormalities, and nutritional status [25]. In this setting, TCR clonality analysis is recommended as an adjunctive tool for subtyping RCeD, particularly for distinguishing RCD-I from RCD-II [36]. Ideally, it should be combined with flow cytometric immunophenotyping of isolated small intestinal IELs; when flow cytometry is unavailable, a combined approach using TCR clonality analysis and immunohistochemistry of duodenal mucosa is recommended [36]. The prognostic significance of isolated TCR clonality in patients with a preserved IEL phenotype remains uncertain and should therefore be interpreted in conjunction with immunophenotypic, histological, and clinical findings.

RCeD type I resembles active uncomplicated CeD and is characterized by phenotypically normal, polyclonal IELs [37,38]. It appears to be more common than RCeD-II, representing approximately 60–90% of RCeD cases [39,40], except of two European studies that found RCeD type II to be more frequent [37,41]. It usually follows a less aggressive clinical course and has a relatively favorable prognosis [37,38]. Clinically, RCeD-I may be difficult to distinguish from slow-responsive CeD, as it lacks a specific aberrant IEL signature, emphasizing the need for careful longitudinal clinical and histological assessment before assigning a refractory phenotype [17].

In contrast, RCeD type II is defined by the expansion of aberrant clonal IELs that lack normal surface T-cell markers and exhibit features of premalignant transformation [42]. These aberrant IELs typically lack surface CD3 and CD8 expression but retain intracellular CD3ε positivity (sCD3^−^/icCD3ε^+^/CD8^−^), and are often quantified by flow cytometry, with ≥20% aberrant IELs commonly supporting the diagnosis [25]. This entity is associated with severe mucosal damage, poor response to conventional therapy, high morbidity, and a markedly increased risk of progression to EATL [40]. RCeD-II may also be associated with severe complications such as ulcerative jejunitis and small-bowel stenosis [43,44]. For this reason, RCeD type II is increasingly regarded as a low-grade intraepithelial lymphoma or an in situ lymphomatous condition [14,45].

**Figure 1:**
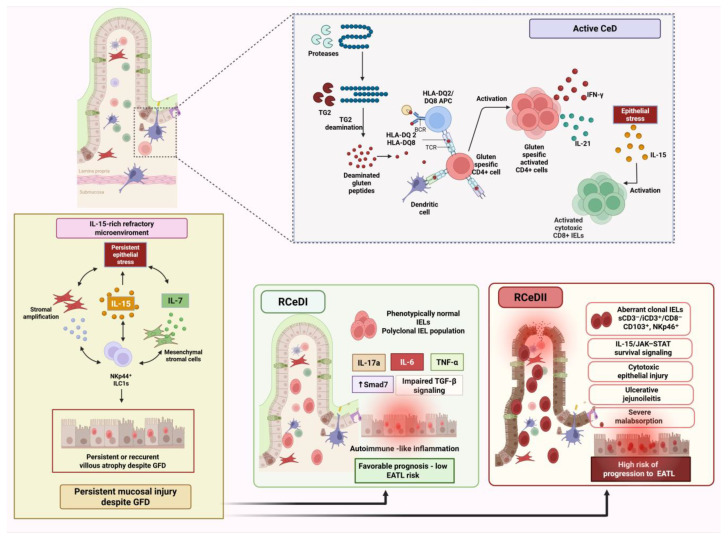
Immune transition from active CeD to refractory disease and classification of RCeD-I and RCeD-II. Persistent villous atrophy despite a strict GFD requires exclusion of ongoing gluten exposure and alternative causes of enteropathy. RCeD-I is characterized by phenotypically normal, polyclonal IELs and persistent immune-mediated injury, whereas RCeD-II involves aberrant clonal IELs, IL-15/JAK–STAT-dependent survival, severe malabsorption, and an increased risk of progression to EATL. Created in Biorender. Aggeletopoulou Ioanna. (2026) https://app.biorender.com/illustrations/6a281932a2393b975d2c1001?slideId=7f47c3c4-8fb3-459e-b883-e796eea4a6c1 (accessed on 10 June 2026). Abbreviations: APC, antigen-presenting cell; CD, cluster of differentiation; CeD, celiac disease; EATL, enteropathy-associated T-cell lymphoma; GFD, gluten-free diet; HLA, human leukocyte antigen; IEL, intraepithelial lymphocyte; IFN-γ, interferon-gamma; IL, interleukin; ILC1, group 1 innate lymphoid cell; JAK, Janus kinase; MICA, MHC class I chain-related protein A; NK, natural killer; NKp44, natural cytotoxicity receptor 2; NKp46, natural cytotoxicity receptor 1; NKG2D, natural killer group 2 member D; RCeD, refractory celiac disease; RCeD-I, refractory celiac disease type I; RCeD-II, refractory celiac disease type II; sCD3, surface CD3; iCD3, intracellular CD3; STAT, signal transducer and activator of transcription; TCR, T-cell receptor; TG2, tissue transglutaminase 2; TGF-β, transforming growth factor-beta; TNF-α, tumor necrosis factor-alpha; Treg, regulatory T cell.

The transition from active CeD to persistent mucosal injury and the subsequent classification into RCeD-I and RCeD-II are summarized in Figure 1.

The main pathogenic, clinical, nutritional, and prognostic variations between RCeD-I and RCeD-II are summarized in Table 1.

Nevertheless, the classification of RCeD into types I and II is based primarily on immunological criteria and may not fully capture the heterogeneity of clinical presentations. Some patients may show clonal or aberrant IEL populations in the absence of severe symptoms, abnormalities in biochemical markers, or persistent histological injuries, creating uncertainty about whether they should be classified and treated as RCeD-II [17]. In such cases, aggressive treatment may not be justified at the time of diagnosis, but close longitudinal monitoring may instead be preferable, as clonal IEL populations may remain stable or even regress over time [17].

The biological boundary between RCeD type II and EATL remains difficult to define, supporting the concept of a continuum from chronic gluten-driven inflammation to aberrant IEL expansion and overt lymphomagenesis [46,47]. Recent advances have highlighted the central role of innate immune activation, epithelial stress signals, interleukin-15–driven survival pathways, cytotoxic intraepithelial lymphocyte activation, and genetic alterations in the progression from uncomplicated CeD to refractory disease and lymphoma [29,48,49]. Understanding these mechanisms is essential not only for refining the diagnosis and classification of RCeD but also for identifying novel therapeutic targets.

From a clinical perspective, persistent villous atrophy despite confirmed adherence to a strict GFD should prompt evaluation for alternative causes of enteropathy, followed by IEL immunophenotyping and TCR clonality analysis to distinguish RCeD-I from RCeD-II [3,36,50]. At the initial diagnosis of RCeD-II, small bowel imaging with capsule endoscopy and computed tomography or magnetic resonance enterography is recommended to exclude ulcerative jejunoileitis and EATL [3,50]. Open-capsule budesonide is recommended as first-line therapy for RCeD-I and may also be used in mild-to-moderate RCeD-II, together with appropriate nutritional support [3,50]. Management of RCeD-II and follow-up of both subtypes should be multidisciplinary and individualized according to disease severity, treatment response, and the risk of complications [3,50].

## 4. Predisposing Factors and Mucosal Microenvironment in RCeD Development

RCeD develops in a highly selected subgroup of patients with CeD and should be considered as the consequence of persistent mucosal immune activation rather than simple dietary treatment failure. Several clinical features appear to contribute to a clinical setting that predisposes patients to develop RCeD. These include older age at CeD diagnosis [51], classical symptomatic presentation with weight loss, diarrhea, abdominal pain, anemia or vitamin deficiencies [52,53], primary unresponsiveness to GFD [54], delayed diagnosis [55], and prolonged gluten exposure [17]. These observations support the concept that long-standing antigenic stimulation may progressively destabilize mucosal immune control. In addition, nutritional deterioration in CeD should not be viewed only as a consequence of mucosal injury but also as a clinical marker of sustained immune activation and delayed restoration of epithelial homeostasis.

Genetic susceptibility may further increase this risk; Al-Toma et al. showed that HLA-DQ2 homozygosity was significantly enriched in patients with RCeD-II and EATL but not in RCeD-I [56]. In their cohort, HLA-DQ2 homozygosity was present in 44.1% of RCeD-II and 53.3% of EATL patients, compared with 20.7% of uncomplicated CeD and 25.5% of RCeD-I [56]. This corresponded to an approximately three-fold increased risk for RCeD-II and four-fold increased risk for EATL, whereas the association with RCeD-I was not significant [56]. Hrdlickova et al. identified a non-HLA genetic locus associated with progression from CeD to RCeD-II [57]. Rs2041570 at chromosome 7p14.3 was independently associated with RCeD-II progression, but not with CeD susceptibility [57]. The risk allele was linked to lower FAM188B expression and genotype-dependent alterations in innate antibacterial and Paneth-cell-related genes, suggesting that non-HLA, non-gluten-dependent epithelial innate immune pathways may also contribute to RCeD-II development [57]. Mechanistically, this association may reflect an HLA-DQ2 gene-dose effect, since HLA-DQ2 homozygous antigen-presenting cells have been shown to induce stronger gluten-specific T-cell proliferation and cytokine secretion than HLA-DQ2/non-DQ2 heterozygous cells [58]. Thus, HLA-DQ2 homozygosity may amplify gluten-driven immune activation and favor progression toward the aberrant IEL/EATL pathway rather than RCeD in general [56,58].

In addition, viral infections have been implicated in CeD onset and exacerbation through disruption of oral tolerance and enhancement of pro-inflammatory immune responses [59]. Although viral infections have not been established as direct drivers of RCeD, viral or viral-like innate immune activation may contribute to a pro-inflammatory intestinal milieu through type I interferon (IFN), toll-like receptor 3 (TLR3)-mediated IL-15 induction, TG2 activation, and disruption of oral tolerance [60,61]. In support of this concept, gliadin-derived innate immune activation may also interact with viral-sensing pathways. Specifically, the P31–43 peptide has been shown to induce inflammation through myeloid differentiation primary response 88 (MyD88) and type I IFN signaling, while its effects are enhanced by poly I:C, supporting a role for TLR3-related mechanisms in amplifying gliadin-induced inflammation [62]. These mechanisms may be particularly relevant in non-responsive CeD and could theoretically sustain IEL activation.

Beyond viral sensing pathways, additional innate and microenvironmental triggers may also induce mucosal inflammation. Non-gluten wheat components, such as α-amylase/trypsin inhibitors, can activate monocytes, macrophages, and dendritic cells through the TLR4–myeloid differentiation factor 2 (MD-2)–cluster of differentiation 14 (CD14) complex [63]. Microbiota-derived proteases and altered epithelial barrier regulation may also modulate gluten processing, enhance inflammatory signaling, and contribute to gluten-independent mucosal activation [64]. These mechanisms should be viewed as additional factors that amplify refractory inflammation rather than established primary causes of RCeD.

## 5. RCeD-I: Persistent Gluten-Independent Inflammatory/Autoimmune-like Pathway

RCeD-I appears to represent a persistent inflammatory phenotype rather than a clonal lymphoproliferative disorder. Its pathogenesis remains incompletely understood and probably encompasses heterogeneous mechanisms of refractoriness to the GFD [30]. Unlike RCeD-II, RCeD-I is characterized by phenotypically normal, polyclonal IELs and resembles active uncomplicated CeD clinically and histologically [45]. A leading hypothesis is that, in some patients, the intestinal immune response shifts from gluten-induced inflammation toward gluten-independent autoimmune-like mucosal injury. This concept is supported by the analysis of duodenal mucosal cytokines by Caruso et al., which showed that the inflammatory profile of RCeD differs from that of active gluten-driven CeD [65]. Specifically, IFN-γ and IL-21, two T helper 1 (Th1)-associated cytokines typically increased in active CeD, were not elevated in RCeD compared with controls. In contrast, increased IL-17A, IL-6, and tumor necrosis factor-alpha (TNF-α) were reported in RCeD, including patients with RCeD-I [65]. These findings suggest that alternative inflammatory pathways may contribute to persistent mucosal injury once classical gluten-dependent Th1 activation is no longer dominant [65].

In addition, defective counter-regulatory signaling may also contribute to persistent mucosal inflammation in RCeD [66]. Sedda et al. showed that Smad7, an intracellular inhibitor of transforming growth factor-beta 1 (TGF-β1) signaling, was increased at the protein level in duodenal biopsies from patients with RCeD, whereas Smad7 RNA was not increased, suggesting post-transcriptional regulation [66]. Increased Smad7 expression was observed in both epithelial and lamina propria compartments and was associated with reduced phosphorylated Smad2/3 despite unchanged active TGF-β1 levels. Functionally, Smad7 knockdown in RCeD biopsy cultures reduced IL-6 and TNF-α expression, indicating that impaired TGF-β1/Smad signaling may enhance the inflammatory cytokine response in RCeD [66]. These findings further support the concept that refractory mucosal injury may be sustained by gluten-independent inflammatory amplification and defective immune regulation.

This interpretation is supported by the association of RCeD-I with other autoimmune disorders and by its generally favorable response to immunosuppressive treatment. IL-15 may also contribute to persistent mucosal inflammation by promoting IEL activation, survival, and cytotoxicity, although its pathogenic role is much more clearly established in RCeD-II than in RCeD-I [67]. Taken together, these findings suggest that RCeD-I represents a sustained immune-mediated mucosal inflammatory reaction in which gluten is no longer the only driver of inflammation, but without the aberrant clonal IEL expansion that defines RCeD-II.

## 6. Epithelial Stress, Stromal Amplification, and Cytotoxic IEL Activation

Although gluten-specific CD4^+^ T-cell responses are essential for CeD initiation, epithelial injury requires additional downstream activation of cytotoxic CD8^+^ IELs, which play a critical role in villous damage [68]. In this context, IL-15 occupies a central position in epithelial injury by activating cytotoxic CD8^+^ IELs, lowering their activation threshold, and upregulating natural killer group 2 member D (NKG2D), thereby promoting TCR-independent killing of stressed enterocytes [69]. Through NKG2D, activated IELs can kill MHC class I chain-related protein A (MICA)-expressing enterocytes [70]. At the same time, IL-15 antagonizes TGF-β1- and T regulatory (Treg) cell-mediated immunosuppression, allowing cytotoxic responses to persist [71,72]. Experimental models further support this mechanism, showing that IL-15-rich mucosal environments require both antigen-specific CD4^+^ T-cell responses and cytotoxic CD8^+^ IEL activation to produce villous atrophy [73,74]. Gliadin-derived peptides may further amplify this epithelial stress response [75]; the P31–43 peptide induces innate immune activation, stimulates IL-15 production, promotes enterocyte apoptosis, and perpetuates epithelial IL-15/IL-15Rα trans-presentation through disruption of epithelial endocytic trafficking [76,77,78]. Sustained IL-15 signaling may therefore induce epithelial remodeling, crypt hyperplasia, and stimulation of cytotoxic innate lymphocytes [67].

Beyond epithelial and IEL-intrinsic mechanisms, innate lymphoid and stromal compartments may further amplify mucosal injury. Intraepithelial ILC1s undergo a phenotypic shift in active CeD and RCeD-I, characterized by a reduction in NKp44^+^ ILCs and an increase in NKp44^−^ cytotoxic ILC1s associated with enhanced IFN-γ expression, cytotoxic potential, and the severity of mucosal damage [79]. In parallel, stromal–immune crosstalk may generate chemokine- and cytokine-rich niches that support the recruitment and retention of cytotoxic CD8^+^ IELs and activated CD4^+^ T cells [80]. Of particular interest, mesenchyme-derived IL-7 has been shown to promote CD8^+^ IEL cytotoxicity before NKG2C/NKG2D upregulation and to trigger epithelial apoptosis independently of gluten exposure [81]. Although much of this evidence derives from active CeD models, it provides an important framework for understanding how epithelial stress, stromal signals, and IL-15-rich mucosal environments may sustain cytotoxic IEL activation when gluten-dependent immune activation is no longer the only driver, a process that becomes pathologically amplified in RCeD-II.

## 7. RCeD-II: Origin, Phenotype, and Survival of Aberrant Intraepithelial Lymphocytes

RCeD-II represents a biologically distinct entity from RCeD-I and is now regarded as a low-grade intraepithelial lymphoma rather than a purely inflammatory complication of CeD [14,45]. In this setting, resistance to the GFD results from progressive epithelial infiltration by aberrant cytotoxic IELs, which are associated with severe malnutrition and a high risk of progression to overt lymphoma. Thus, in RCeD-II, nutritional failure reflects not only impaired absorption but also the biological aggressiveness of the underlying intraepithelial lymphoproliferative process. Diagnosis therefore relies on the demonstration of a clonal population of neoplastic IELs with an atypical immunophenotype [82].

Phenotypically, RCeD-II is characterized by the expansion of aberrant IELs that typically lack surface CD3, surface TCR, CD8, CD19, and CD56, while retaining intracellular/cytoplasmic CD3 positivity and clonal TCR gene rearrangement [83,84]. This profile suggests early T-cell commitment with incomplete T-cell differentiation and acquisition of natural killer (NK)-like characteristics, including expression of NK receptors such as NKp46 [83,84]. By multicolor flow cytometry, these cells are commonly identified within the CD103^+^ IEL compartment as sCD3^−^/iCD3^+^/CD8^−^/αβTCR^−^ cells [85], with a 20% cutoff of aberrant IELs proposed to distinguish RCeD-II from RCeD-I [36,50,85]. Immunohistochemistry is less precise because cytoplasmic and membranous CD3 cannot be reliably distinguished in paraffin sections; therefore, higher cutoffs are generally required when this method is used [14]. NKp46 staining has also emerged as a useful marker, with increased NKp46^+^ IELs helping to discriminate RCeD-II from RCeD-I [86].

TCR gene clonality analysis by multiplex PCR, usually performed on DNA extracted from fresh or paraffin-embedded duodenal biopsies, can further support the diagnosis of RCeD-II [87]. However, clonality should be interpreted as an adjunctive finding because clonal or oligoclonal TCR rearrangements may also be detected in RCeD-I and, less frequently, in uncomplicated CeD [87]. Rearrangement analysis of γ TCR chain (TRG) is commonly preferred for diagnostic assessment, whereas additional β TCR chain (TRB) or δ TCR chain (TRD) testing may be useful in selected cases, particularly when TRG results are negative or when further characterization of the aberrant IEL population is required [30]. Thus, aberrant IELs display a hybrid T/NK phenotype rather than the profile of conventional activated gluten-specific T cells.

Emerging evidence links aberrant IELs in RCeD-II/EATL to innate-like lymphocytes present in the normal gut mucosa [26,88]. These cells are proposed to originate from immature hematopoietic precursors that enter the intestinal epithelium and initiate T-cell differentiation under NOTCH1 signaling [26]. In an IL-15-rich microenvironment, however, full T-cell differentiation is interrupted, and the cells are reprogrammed toward an NK-like phenotype [26]. In this model, TGF-β promotes acquisition of CD103, whereas IL-15 favors expression of NK-cell markers and cytotoxic features [26]. Consequently, these innate-like IELs retain T-cell-associated characteristics, such as intracellular CD3 positivity and TCR gene rearrangements, while also expressing NK-cell-associated markers, including NKp46 [26].

The origin of these aberrant IELs remains debated. Schmitz et al. identified a rare lineage-negative Lin^−^CD7^+^CD127^−^CD34^−^ IEL population, present in non-refractory duodenal mucosa and responsive to IL-15 through CD122 expression, which may represent the physiological counterpart of the aberrant IELs expanded in RCeD-II [83]. In addition, Tack et al. showed that aberrant IELs display increased granzyme B expression and heterogeneous TCR rearrangement patterns, suggesting cytotoxic differentiation and variable maturation stages, with more mature aberrant T-cell populations potentially associated with higher risk of EATL progression [84].

IL-15 is central to the persistence of aberrant IELs in RCeD-II. In the RCeD-II mucosa, IL-15 overexpression promotes expansion of aberrant IELs primarily through decreased apoptosis and increased survival, rather than through increased proliferation [28,83]. Mechanistically, IL-15 inhibits apoptosis through the Janus kinase 3 (JAK3)/signal transducer and activator of transcription 5 (STAT5) pathway and induction of the anti-apoptotic molecule Bcl-xL [27]. This survival pathway allows aberrant IELs to accumulate within the intestinal epithelium despite their low proliferative index.

At the functional level, expanded aberrant IELs are highly cytotoxic. Enterocyte-derived IL-15 may activate these cells and promote cytotoxic responses against epithelial cells, contributing to the severe enteropathy observed in RCeD-II [28]. This cytotoxic epithelial attack provides a mechanistic explanation for the extensive mucosal damage, ulcerative jejunoileitis and severe malabsorptive phenotype that distinguish RCeD-II from RCeD-I [28].

Finally, this model also connects aberrant IEL origin and survival with lymphomagenesis, as iCD3^+^ innate IELs in RCeD-II/EATL may acquire gain-of-function mutations in JAK1 or STAT3, enhancing IL-15 responsiveness and promoting clonal expansion [26]. This genetic evolution provides a mechanistic bridge between RCeD-II and EATL.

## 8. Cytotoxic Epithelial Injury and Dissemination of Aberrant IELs

The accumulation of aberrant IELs in RCeD-II has detrimental consequences for epithelial integrity [28]. In RCeD-II, IL-15-driven survival of aberrant IELs is accompanied by marked cytotoxic activity, and expanded aberrant IELs can mediate an intense cytolytic attack against the intestinal epithelium [28]. Recent functional evidence indicates that this epithelial injury is largely granzyme-B dependent [89]. Castelijn et al. showed that granzyme-B expression is significantly upregulated in aberrant IELs from patients with RCeD-II compared with CeD patients on a GFD and correlates with the severity of villous atrophy and clinical response to therapy [89]. In the presence of enterocytes, aberrant IELs degranulate and secrete granzyme-B, leading to enterocyte apoptosis mainly through activation of the intrinsic apoptotic pathway [89]. Importantly, this cytotoxic process requires direct cell–cell contact and is mediated by CD103–β7-dependent binding, rather than NKG2D-dependent killing. Mechanistically, this CD103–β7 axis may promote lytic granule polarization and exocytosis after binding to epithelial E-cadherin, allowing granzyme-B delivery to enterocytes [90]. IL-15 may further amplify this cytotoxic program through STAT5-mediated induction of granzyme-B transcription, thereby linking the survival and effector functions of aberrant IELs [91,92]. Blocking CD103 reduced degranulation, granzyme-B secretion, and epithelial cell death, while β7 blockade with etrolizumab prevented enterocyte killing and preserved human intestinal organoid viability in a preclinical model [89]. These findings provide a mechanistic explanation for the severe mucosal injury, ulcerative jejunoileitis, and extreme malabsorptive phenotype observed in RCeD-II [28].

Clinically and histologically, this cytotoxic process distinguishes RCeD-II from RCeD-I. RCeD-II is associated with more extensive intestinal involvement, worse malabsorption, hypoalbuminemia, severe mucosal damage, and a higher frequency of ulcerative jejunitis than RCeD-I [30]. Malamut et al. reported ulcerative jejunitis in 67.4% of patients with RCeD-II compared with 28.6% of patients with RCeD-I [37]. Similarly, Barret et al. showed by capsule endoscopy that distal small-bowel lesions, involving the distal jejunum and ileum, were more frequently observed in RCeD-II patients than in RCeD-I patients, occurring in 54% and 9% of cases, respectively [93]. In line with this evidence, other reports indicate that symptom burden is greater in RCeD-II because of extensive bowel involvement [94] and mucosal ulcerations [40].

The tissue distribution of aberrant IELs is not limited to the duodenal epithelium. Cells sharing the aberrant IEL phenotype have been identified in the lamina propria, additional gastrointestinal epithelial sites, peripheral blood, skin, lungs, and additional organs [42,95,96,97,98]. Similar abnormal populations may also be present in lymphocytic gastritis and lymphocytic colitis, which have been reported in approximately 30–50% of patients with RCeD-II [37], indicating that the aberrant IEL compartment can extend beyond the small intestinal mucosa [42,97]. This dissemination may explain why EATL does not always arise within the small intestine but may also present at extraintestinal sites, including the skin, lung, lymph nodes, bone marrow, or other organs [42,95,96,97,98]. Thus, RCeD-II should not be considered merely as a localized enteropathy but as a disseminated intraepithelial lymphoproliferative process with potential progression to intestinal or extraintestinal EATL [99].

Taken together, cytotoxic epithelial injury and tissue dissemination represent two connected features of RCeD-II biology. Locally, aberrant IELs drive severe epithelial damage, ulcerative jejunoileitis, and malabsorption through cytotoxic mechanisms, including granzyme-B-dependent epithelial killing [28,89]. Beyond the duodenal epithelium, the detection of phenotypically similar aberrant IELs in other gastrointestinal and extraintestinal sites supports the concept that RCeD-II is a disseminated intraepithelial lymphoproliferative process with potential progression to intestinal or extraintestinal EATL [30].

## 9. Clonal Evolution and the RCeD-II–EATL Continuum

RCeD-II is best understood as a clonal lymphoproliferative disorder that lies on the biological continuum between refractory enteropathy and overt EATL [14,46,82]. This concept is supported by the current view of RCeD-II as a low-grade intraepithelial lymphoma or in situ lymphoma, rather than as a purely inflammatory complication of CeD [14,46,82]. In this setting, resistance to the GFD reflects the persistence and expansion of aberrant cytotoxic IELs with malignant potential.

Although the neoplastic nature of RCeD-II IELs has long been debated because these cells may show relatively normal cytology and low proliferative activity, several biological features support their malignant or pre-malignant status [40]. The disseminated distribution of aberrant IELs, described above, further supports their neoplastic potential. Moreover, clonal TCR rearrangements reported in IELs of patients with RCeD-II may also be found in EATL arising in the same disease context, indicating that they originate from a common clonal precursor [84,99]. In addition, aberrant IELs in RCeD-II and EATL share overlapping phenotypic features, chromosomal abnormalities, and oncogenic somatic mutations [46,48,100,101]. Together, these findings support the interpretation that aberrant IELs in RCeD-II represent precursor cells of EATL and place RCeD-II within a biological continuum between refractory enteropathy and overt lymphoma.

The molecular basis of this progression is strongly linked to dysregulated JAK/STAT signaling [102]. Somatic gain-of-function mutations in JAK1 or STAT3 are among the most recurrent genetic events in RCeD-II and EATL, being detected in approximately 80% of RCeD-II cases and 90% of EATL cases [46]. Among JAK/STAT alterations, mutations affecting JAK1 glycine 1097 represent a recurrent hotspot event, reported in approximately 50% of RCeD-II patients and in 68% of EATL cases complicating RCeD-II [46]. This hotspot involves a highly conserved residue located at the interaction site between JAK1 and suppressor of cytokine signaling 1 (SOCS1), a negative regulator of JAK1 activity, thereby potentially impairing SOCS1-mediated inhibition and enhancing JAK/STAT signaling [103]. STAT3 mutations, present in approximately 30–40% of cases, occur mainly at Src homology 2 (SH2)-domain hotspots and enhance STAT3 activation downstream of JAK1 [26,46].

Because the JAK1–STAT3 pathway regulates lymphocyte proliferation, survival, and activation, these mutations are thought to provide a competitive growth or survival advantage to aberrant IELs within the chronically inflamed celiac mucosa. However, JAK1/STAT3 activation per se does not fully explain transformation to EATL. Additional molecular lesions appear to cooperate with cytokine-driven survival pathways and promote further clonal evolution. These include alterations in negative regulators of JAK/STAT signaling, such as SOCS1, SOCS3, and SH2B3 [46,48]. Other recurrent events affect TNFAIP3, a negative regulator of NF-κB signaling, either through mutation or chromosomal loss [46,48]. Additional alterations involve epigenetic regulators, including TET2 and KMT2D [46,48]. Trisomy 1q has also been described as a recurrent event in RCeD-II IELs and may contribute to transformation through effects on cell-cycle and tumor-suppressor pathways [46,48,104].

Together, these findings support a stepwise model of RCeD-II lymphomagenesis. IL-15-rich mucosal inflammation promotes survival and accumulation of aberrant IELs, while JAK1/STAT3 gain-of-function mutations and related pathway alterations provide a selective advantage to these cells [30]. Subsequent genetic events may then drive progression from an intraepithelial clonal disorder toward invasive lymphoma [30]. In line with this model, longitudinal genetic analyses showed that RCeD-II samples generally maintained stable mutation profiles, whereas subsequent EATLs acquired additional mutations and were clonally related to the antecedent RCeD-II lesions [48]. Although the functional contribution of each acquired alteration remains uncertain, the emergence of new variants in transformed EATL supports a model of clonal evolution from RCeD-II toward overt lymphoma [48].

Recent single-cell multiomic evidence has further refined this model [105]. Singh et al. analyzed duodenal biopsies from patients with RCeD-I, RCeD-II, active newly diagnosed CeD, and non-CeD controls and showed that the aberrant sCD3^−^ lymphocytes defining RCeD-II harbor lymphoma-driver somatic mutations, display features of an innate lymphoid cell/progenitor T-cell–like state, and undergo extensive TCR (TRA, TRB, and TRD) recombination [105]. Importantly, the same study identified sCD3^+^ T cells carrying lymphoma-driver mutations in 6 of 10 patients with RCeD-I; these mutant cells formed large TCRαβ clones and displayed inflammatory and cytotoxic molecular profiles [105]. These findings suggest that RCeD may involve a broader spectrum of genetically altered intestinal lymphocyte clones, ranging from mutated cytotoxic/inflammatory sCD3^+^ T-cell clones in RCeD-I to highly expanded aberrant sCD3^−^ progenitor-like clones in RCeD-II, thereby strengthening the biological link between chronic nonresponsive enteropathy, clonal lymphocyte evolution, and progression toward EATL [105].

In addition, more recent single-cell evidence showed that malignant RCeD-II IELs display substantial intratumoral heterogeneity despite shared transcriptional features across patients [106]. This heterogeneity increased with tumor burden, clinical severity, and progression toward EATL, supporting a model of subclonal diversification during RCeD-II evolution [106]. Importantly, this study identified a proliferative RCeD-II tumor-cell cluster expressing MKI67 and STMN1, which was present even in low-burden samples and, by trajectory analyses, appeared to seed other tumor-cell states, including states observed after EATL onset [106]. These findings suggest that RCeD-II progression may be driven not only by JAK/STAT activation but also by dynamic intratumoral evolution of proliferative subclones [106].

From a pathological perspective, these molecular and clonal data reinforce the view that RCeD-II and EATL should not be regarded as entirely separate disease processes [45,48]. Rather, RCeD-II–associated lymphomagenesis is best interpreted as a continuous process of clonal evolution, in which chronic mucosal inflammation, IL-15–driven IEL survival, clonal expansion, and progressive acquisition of oncogenic mutations converge to sustain aberrant IELs as an intraepithelial lymphomatous population [45]. Within this spectrum, overt EATL should be considered when the neoplastic lymphoid population extends beyond the intraepithelial compartment and extensively infiltrates the duodenal or small-intestinal wall, particularly in the presence of a tumor mass, perforation, or stricture formation [45]. Thus, EATL may be viewed as the invasive stage of the RCeD-II disease process, with both conditions sharing a common celiac-associated background and recurrent molecular alterations, especially in pathways involving JAK/STAT and NF-κB signaling [45,48]. Recent comparative molecular profiling of intestinal T-cell lymphomas further supports the distinct molecular identity of EATL, showing that JAK/STAT pathway mutations in EATL preferentially involve JAK1 and STAT3, together with recurrent alterations in epigenetic regulators such as TET2, ARID1A, and KMT2D [107]. These findings reinforce the relevance of the JAK1/STAT3 axis and epigenetic dysregulation in celiac-associated lymphomagenesis.

**Figure 2:**
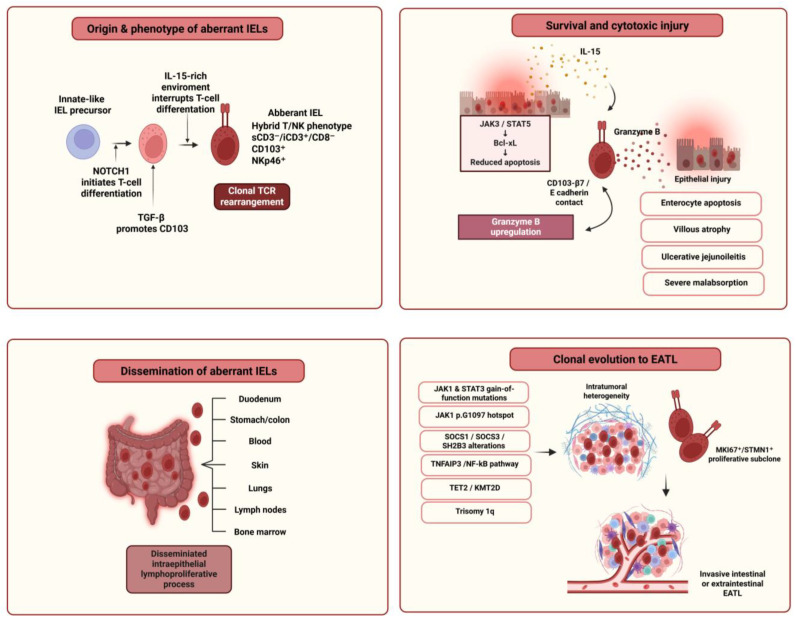
RCeD-II pathogenesis and the RCeD-II–EATL continuum. RCeD-II is characterized by aberrant clonal IELs with a hybrid T/NK phenotype, typically sCD3^−^/iCD3^+^/CD8^−^, CD103^+^, and NKp46^+^. In an IL-15-rich microenvironment, JAK3/STAT5 and Bcl-xL-dependent signaling promotes their survival, while CD103–β7/E-cadherin-mediated contact and granzyme B release contribute to epithelial apoptosis, villous atrophy, ulcerative jejunoileitis, and severe malabsorption. Aberrant IELs may disseminate beyond the duodenal epithelium, supporting the concept of RCeD-II as a disseminated intraepithelial lymphoproliferative disorder. Progression toward EATL involves clonal expansion, JAK1/STAT3 gain-of-function mutations, dysregulation of JAK/STAT and NF-κB signaling, epigenetic alterations, trisomy 1q, and increasing intratumoral heterogeneity, ultimately leading to invasive lymphoma. Created in Biorender. Aggeletopoulou Ioanna. (2026) https://app.biorender.com/illustrations/6a281932a2393b975d2c1001?slideId=7f47c3c4-8fb3-459e-b883-e796eea4a6c1 (accessed on 10 June 2026). Abbreviations: Bcl-xL, B-cell lymphoma-extra-large; CD, cluster of differentiation; EATL, enteropathy-associated T-cell lymphoma; IEL, intraepithelial lymphocyte; IL, interleukin; JAK, Janus kinase; MKI67, marker of proliferation Ki-67; NF-κB, nuclear factor-kappa B; NK, natural killer; NKp46, natural cytotoxicity receptor 1; RCeD-II, refractory celiac disease type II; sCD3, surface CD3; iCD3, intracellular CD3; SOCS, suppressor of cytokine signaling; STAT, signal transducer and activator of transcription; STMN1, stathmin 1; TCR, T-cell receptor; TET2, ten-eleven translocation methylcytosine dioxygenase 2; TGF-β, transforming growth factor-beta.

Overall, RCeD- lymphomagenesis can be viewed as a multistep, although not necessarily strictly linear, process. Epithelial IL-15 sustains the survival and accumulation of aberrant IELs through JAK3/STAT5-dependent anti-apoptotic signaling, while these cells also mediate cytotoxic epithelial injury. Activating JAK1/STAT3 mutations provide a selective advantage, whereas additional alterations involving JAK/STAT regulators, NF-κB signaling, epigenetic regulators, and chromosome 1q may cooperate in clonal evolution. The acquisition of further molecular alterations and increasing subclonal heterogeneity may ultimately facilitate progression to overt EATL, as summarized in Figure 2.

## 10. Future Directions and Conclusions

Recent advances have substantially improved our understanding of RCeD as a spectrum of immune-mediated and lymphoproliferative disorders rather than a uniform complication of CeD. RCeD-I appears to reflect persistent gluten-independent mucosal inflammation with a preserved IEL phenotype, whereas RCeD-II is driven by the survival, expansion, cytotoxic activation, and clonal evolution of aberrant IELs. Central to this process is the IL-15/JAK–STAT axis, which promotes IEL persistence and cytotoxicity, while recurrent genetic alterations, particularly involving JAK1, STAT3, TNFAIP3, TET2, KMT2D, and related pathways, provide a molecular basis for progression toward EATL. Single-cell multiomic studies have further expanded this model by showing that clonal lymphocyte evolution may occur across refractory CeD and that RCeD-II contains heterogeneous and potentially treatment-resistant tumor-cell subpopulations.

Future research should focus on prospective molecular monitoring of patients with non-responsive and refractory CeD, integrating IEL immunophenotyping, TCR clonality, molecular profiling, and single-cell approaches to improve early risk stratification. Candidate parameters for evaluation include serial assessment of aberrant IEL phenotype and burden together with TCR clonality, as well as exploratory targeted profiling of recurrent JAK1/STAT3 alterations, such as JAK1 p.G1097 and STAT3 SH2-domain mutations. Such strategies may help distinguish patients with stable clonal IEL populations from those at increased risk of progression to EATL. Therapeutically, targeting IL-15/JAK–STAT signaling remains biologically attractive, but recent evidence also indicates that pathway inhibition alone may not eliminate malignant clones and may select resistant subpopulations. Therefore, future approaches will probably require molecularly informed combination strategies aimed not only at suppressing inflammation but also at preventing aberrant IEL survival, clonal expansion, epithelial cytotoxicity, and lymphoma transformation. Future clinical trials should include well-characterized patients stratified by RCeD subtype, aberrant IEL burden, and molecular profile and should assess histological response, nutritional status, changes in aberrant IEL burden, and EATL-free or progression-free survival. From the perspective of nutritional immunology, RCeD demonstrates that dietary antigen exclusion may be insufficient once autonomous mucosal immune activation and clonal IEL evolution have been established. Future management should therefore integrate strict dietary assessment, correction of nutritional deficiencies, appropriate nutritional support, immune profiling, and molecular monitoring to personalize care and reduce the risk of progression. Overall, understanding the immune pathogenesis of RCeD provides the basis for earlier diagnosis, individualized monitoring, and more rational targeted interventions in this high-risk complication of CeD.

## Figures and Tables

**Table 1:** Comparative pathogenic, clinical, and nutritional features of RCeD-I and RCeD-II.

Characteristic	RCeD-I	RCeD-II
Biological interpretation	- Persistent immune-mediated refractory enteropathy with a preserved IEL phenotype - Generally considered an inflammatory or autoimmune-like form of refractory disease	- Clonal intraepithelial lymphoproliferative disorder - Increasingly regarded as a low-grade intraepithelial lymphoma or in situ lymphomatous condition.
IEL phenotype	- Phenotypically normal IELs, resembling those observed in active uncomplicated CeD	- Aberrant IELs with loss of normal surface T-cell markers, typically sCD3^−^/icCD3ε^+^/CD8^−^, frequently identified within the CD103^+^ IEL compartment
TCR clonality	- Usually polyclonal - However, clonal or oligoclonal TCR rearrangements may occasionally be detected and should be evaluated in the broader diagnostic context	- Usually associated with clonal TCR rearrangement, particularly when combined with an aberrant IEL phenotype, persistent villous atrophy, and clinical severity.
Dominant pathogenic process	- Persistent gluten-independent mucosal inflammation - Defective immune regulation - Cytokine-mediated epithelial injury without overt lymphoproliferative transformation	- IL-15/JAK–STAT–driven survival of aberrant IELs - Cytotoxic epithelial injury - Clonal expansion - Progressive molecular evolution toward lymphoma
Clinical course	- Usually less aggressive - May overlap clinically with slow-responsive CeD	- More aggressive disease course - Often associated with severe mucosal damage, malabsorption, poor response to conventional therapy, and higher morbidity
Nutritional profile	- Nutritional deficiencies may occur due to persistent villous atrophy, but severe malnutrition is generally less prominent than in RCeD-II	- Severe malabsorption, weight loss, hypoalbuminemia, anemia, micronutrient deficiencies, and malnutrition are more frequent and may reflect disease severity
Complications	- Lower frequency of severe complications - Careful follow-up is necessary	- Higher risk of ulcerative jejunoileitis, small-bowel stenosis, extensive intestinal involvement, dissemination of aberrant IELs, and progression to EATL
Prognosis	- Generally, more favorable, particularly when nutritional status is preserved and no aberrant IEL population is detected	- Poorer prognosis due to severe nutritional deterioration, lymphoproliferative biology, and increased risk of transformation to EATL
EATL risk	- Low, although long-term monitoring is important	- Markedly increased - RCeD-II is considered part of the biological continuum linking refractory enteropathy with EATL

Abbreviations: CeD, celiac disease; EATL, enteropathy-associated T-cell lymphoma; IELs, intraepithelial lymphocytes; RCeD, refractory celiac disease; TCR, T-cell receptor.

## Data Availability

No new data were created or analyzed in this study. Data sharing is not applicable to this article.

## Abbreviations

anti-TG2/anti-tTG, anti-tissue transglutaminase 2 antibodies; CD14, cluster of differentiation 14; CeD, celiac disease; EATL, enteropathy-associated T-cell lymphoma; EMA, anti-endomysial antibodies; GFD, gluten-free diet; HLA, human leukocyte antigen; icCD3ε, intracellular/cytoplasmic CD3 epsilon; IEL, intraepithelial lymphocyte; IFN, interferon; IgG anti-DGP, immunoglobulin G anti-deamidated gliadin peptide antibodies; IL-15, interleukin-15; JAK3, Janus kinase 3; MD-2, myeloid differentiation factor 2; MICA, major histocompatibility complex class I chain-related protein A; MyD88, myeloid differentiation primary response 88; NK, natural killer; NKG2D, natural killer group 2 member D; NRCeD, non-responsive celiac disease; RCeD, refractory celiac disease; RCeD-I, refractory celiac disease type I; RCeD-II, refractory celiac disease type II; sCD3, surface CD3; SH2, Src homology 2; SOCS1, suppressor of cytokine signaling 1; STAT5, signal transducer and activator of transcription 5; TCR, T-cell receptor; TGF-β1, transforming growth factor-beta 1; Th1, T helper 1; TLR3, toll-like receptor 3; TNF-α, tumor necrosis factor-alpha; TRB, T-cell receptor beta chain; TRD, T-cell receptor delta chain; TRG, T-cell receptor gamma chain; Treg, T regulatory cell.
